# Testing the Associations Between Body Image, Social Support, and Physical Activity Among Adolescents and Young Adults Diagnosed With Cancer

**DOI:** 10.3389/fpsyg.2021.800314

**Published:** 2022-01-03

**Authors:** Madison F. Vani, Catherine M. Sabiston, Linda Trinh, Daniel Santa Mina

**Affiliations:** Faculty of Kinesiology and Physical Education, University of Toronto, Toronto, ON, Canada

**Keywords:** body image, cancer, adolescents, young adults, physical activity, social support, quantitative, oncology

## Abstract

Physical activity (PA) is important for managing the side effects and long-term outcomes of cancer treatment, yet many adolescents and young adults diagnosed with cancer (AYAs) are not meeting PA guidelines. Body image and social support are two factors that can influence PA behavior and require further attention in this population. The purpose of this study was to examine the associations between body image, social support, and PA among AYAs. An online cross-sectional survey administered through the Research Electronic Data Capture platform was used to assess self-reported body image (body-related self-conscious emotions of appearance and fitness shame, guilt, authentic pride, and hubristic pride), social support (general and cancer-specific), and PA (mild, moderate-to-vigorous, and resistance exercise) in AYAs (*N* = 119; *M*_age_ = 34.5 ± 5.5 years). Based on findings from path analyses, body image and social support were directly associated with PA (*R*^2^ = 0.09–0.33). Social support was also directly associated with body image. However, there were no indirect effects. These findings provide preliminary support for the influential role of appearance and fitness body-related emotions and cancer-specific social support on PA. The results have important implications for the development of targeted strategies aimed at improving body image (e.g., cognitive dissonance and compassion-focused interventions) and social support (e.g., facilitating the provision of cancer-specific support), with the overall goal of increasing AYAs’ PA.

## Introduction

Cancer in adolescents and young adults (AYAs; diagnosed between 15 and 39 years) accounts for approximately 5% of all new cases of cancer diagnosed each year in North America (American Cancer Society, 2019; Canadian Cancer Statistics Advisory Committee, 2019). With improvements in early screening and treatment, many AYAs are living 50–60 years after diagnosis (Lewis et al., 2014; Canadian Partnership Against Cancer, 2017). However, cancer treatment can have significant acute and long-term effects on AYAs’ physical, psychological, and social health and well-being (Tai et al., 2012; Patterson et al., 2015). Identifying strategies to help offset these detrimental outcomes of cancer is needed.

Physical activity (PA) during and after cancer treatment is associated with many benefits including reduced adverse effects, mortality, and cancer recurrence (Bélanger et al., 2011; Baumann et al., 2013; Cormie et al., 2017; Brunet et al., 2018). The consistent well-documented benefits of PA are reflected in PA guidelines for cancer survivors, with the most recent recommendations described as at least 90 min of aerobic activity and two sessions of resistance exercise per week (Campbell et al., 2019). Yet, about half of AYAs are inactive or insufficiently active, with PA levels declining from pre-diagnosis to during and post-treatment (Bélanger et al., 2011; Murnane et al., 2015). Therefore, it is important to identify potential intervening factors that may help to improve AYAs’ PA levels and overall health and well-being.

Social support is likely an important factor that can affect PA. Social support can be conceptualized as a multidimensional construct that involves interpersonal interactions from support networks (Bianco and Eklund, 2001). Even though AYAs are in a developmental stage marked by fostering social connections, a cancer diagnosis impedes their ability to create and maintain social relationships (Patterson et al., 2015). Social disconnect may undermine perceptions of physical skills and abilities (Feeney and Collins, 2015). AYAs report that perceptions of low social support for PA limit their interest in participating in activity (Wu et al., 2015). Further, AYAs describe that holding discordant PA goals when compared to others challenges their willingness to engage in activity (Wu et al., 2015). Meanwhile, group-based PA increases AYAs’ PA engagement and maintenance (Gill et al., 2016). As such, social support may be an important factor related to PA among AYAs.

Body image is another key factor that can impact PA behavior. Body image is a multidimensional construct that involves positive and negative perceptions, cognitions, feelings, and behaviors toward the body’s appearance and function (Cash and Smolak, 2011). Although appearance has been widely studied in previous research with AYAs, there is a paucity of studies examining functional body image (Vani et al., 2021). Up to 88% of AYAs report changes to their body’s appearance and physical function as a consequence of cancer and related treatment (Vani et al., 2021). Body image theories within oncology (White, 2000) posit that cancer-related body changes (e.g., scarring, hair loss, weight change, and physical functioning issues) can negatively impact body image perceptions, cognitions, affect, and behaviors. Particularly related to body image affect, AYAs have described feelings of self-consciousness, shame, and guilt associated with body changes resulting from cancer (Williamson et al., 2010; Pounders et al., 2017). Self-consciousness is often measured *via* body-related shame and guilt (Castonguay et al., 2014, 2016). Body-related shame occurs when an individual feels they have a flaw related to their sense of self, while body-related guilt occurs when an individual has a negative evaluation of their behavior (Tracy and Robins, 2007). Meanwhile, some AYAs have reported body-related pride, a positive self-conscious emotion (Wallace et al., 2007; Grogan and Mechan, 2017). Pride is experienced when an individual evaluates their engagement in socially valued behaviors or their social presentation of their body in a positive manner (Tracy and Robins, 2007). Pride can be further divided into two facets: authentic (i.e., positively evaluating one’s appearance- or fitness-related achievements) and hubristic (i.e., evaluating body appearance or function as superior to others) pride (Tracy and Robins, 2007; Castonguay et al., 2013, 2014, 2016). Positive and negative appearance- and fitness-related self-conscious emotions have been associated with PA among AYAs who have not been diagnosed with cancer (Sabiston et al., 2010; Castonguay et al., 2015; Gilchrist et al., 2018; Pila et al., 2020), yet these relationships have not been tested among AYAs.

In addition to the plausible direct association between body-related emotions and PA, body image may indirectly influence the relationship between social support and PA. Although these associations are likely to be similar to the general population, the relationships may be more pronounced among AYAs due to the unique social disconnect, body-related changes, and difficulties with PA engagement that AYAs may experience (Murnane et al., 2015; Patterson et al., 2015; Vani et al., 2021). Supportive others are central to helping AYAs feel more positively and less negatively about their bodies and provide protection against negative body commentary from others (Larouche and Chin-Peuckert, 2006; Williamson et al., 2010; Vani et al., 2021). As such, social support may be positively associated with body-related pride and negatively associated with body-related shame and guilt. These associations are consistent with sociocultural perspectives (Petrie and Greenleaf, 2012), wherein social influences (e.g., peers, parents) are often implicated in the development of thoughts and feelings about the body and exercise behaviors. AYAs’ body-related perceptions and feelings have been reported as a barrier to PA engagement after treatment (Wu et al., 2015). Further, AYAs who hold negative body perceptions avoid physical activities that emphasize the body’s appearance (e.g., swimming) and communal changing spaces that are common in fitness facilities (Larouche and Chin-Peuckert, 2006; Vani et al., 2021). Interpreting this evidence, positive body-related emotions may enhance PA participation whereas negative body-related emotions may impede PA participation (Sabiston et al., 2010; Gilchrist et al., 2018; Pila et al., 2020). However, body-related guilt can also be reparative and increase PA (Sabiston et al., 2010; Castonguay et al., 2017). Furthermore, these emotions may be uniquely associated with different PA intensities and types. To promote PA among AYAs, it is important to better understand the associations among social support, body image, and PA after a cancer diagnosis.

Drawing on sociocultural perspectives (Petrie and Greenleaf, 2012) and White’s (2000) body image model in oncology social support and body image may be factors associated with PA and body image may indirectly influence the relationship between social support and PA. The aim of this study was to test the pathways of social support, appearance- and fitness-related self-conscious emotions of shame, guilt, authentic pride, hubristic pride, and PA. Given the PA recommendations for participation in both aerobic and resistance-based exercise, both types of PA were examined. Based on existing literature (Vani et al., 2021) and conceptual underpinnings (Petrie and Greenleaf, 2012), it was expected that social support would be negatively associated with shame and guilt and positively associated with authentic and hubristic pride. Shame was expected to demonstrate a negative relationship with PA. The reparative nature of guilt (Tangney and Dearing, 2002) suggested that guilt would be positively tied to PA. Authentic and hubristic pride and social support were also expected to demonstrate a positive relationship with PA. Finally, it was hypothesized that self-conscious emotions would indirectly influence the relationship between social support and PA.

## Materials and Methods

### Participants and Procedure

Following university research ethics board approval (#39245), AYAs were recruited by emailing cancer organizations and survivorship programs in Canada and the United States. Supporting organizations and programs contacted the AYAs via email and/or social media. A recruitment poster was also shared on the research team’s social media accounts. AYAs were eligible to participate if they (i) were diagnosed with cancer between the ages of 15 and 39 years, regardless of cancer type, stage, time since diagnosis, treatment status, and treatments received; and (ii) were able to read and respond to questions in English. The selected age range is aligned with the standard for cancer organizations in Canada and the United States (American Cancer Society, 2019; Canadian Cancer Statistics Advisory Committee, 2019). Interested eligible AYAs used the provided link or QR code to directly access the electronic consent form. Once consent was complete, participants were redirected to the secure online survey using the Research Electronic Data Capture platform. Upon completion, participants were entered into a draw to win one of twenty $25 gift cards. Surveys were completed between January and June, 2021. Based on this timing, data collection mainly occurred during public health restrictions pertaining to the COVID-19 pandemic. Standard sample size estimate protocols for path analyses suggest 10–20 participants per main parameter (Nunnally, 1967; Stevens, 2012; Kline, 2015). Based on these recommendations, the target sample size was 120 AYAs.

### Measures

#### Demographics

Participants completed a sociodemographic questionnaire containing personal (e.g., age, self-identified gender, sexuality, and ethnicity) and cancer-related (e.g., cancer type, treatment status, and time since diagnosis) questions.

#### Social Support

Social support was measured using the Social Support Survey (Richman et al., 1993), which was modified to include a cancer-specific support type. Participants were provided with the definition of each of the following support types: listening, task challenge, emotional, reality confirmation, tangible assistance, and based on previous literature, a cancer-specific support type (i.e., “people who support you by letting you know that they understand what it is like to have gone through cancer”; McDonough et al., 2014). For each support type, participants completed two items assessing the (i) quantity (i.e., “how many individuals provided you with this type of support”) on a 5-point Likert-type scale from 0 (*none*) to 4 (*8 or more*), and (ii) quality (i.e., “in general, how satisfied are you with the overall quality of support you receive?”) on a 5-point Likert-type scale from 0 (*very dissatisfied*) to 4 (*very satisfied*). A total general social support score was calculated by averaging the quantity and quality items across the five general support types. Aligning with research practice (McDonough et al., 2014), a total cancer-specific social support score was calculated by averaging the quantity and quality items. Evidence of validity and reliability were previously reported with non-cancer AYAs (Richman et al., 1993; Rees et al., 2007) and adult breast cancer survivors (McDonough et al., 2014).

#### Body Image

Body image was operationalized as appearance- and fitness-related self-conscious emotions (Castonguay et al., 2014, 2016).

##### Appearance-Related Self-Conscious Emotions

Participants completed the 16-item Body and Appearance Self-Conscious Emotions Scale (BASES; Castonguay et al., 2014) to assess appearance-related shame, guilt, authentic pride, and hubristic pride (four-items per emotion). Participants were asked to indicate how often, on average, they experienced appearance-related shame (e.g., “ashamed of the way I look”), guilt (e.g., “guilty that I do not do enough to improve the way I look”), authentic pride (e.g., “proud of the effort I place on maintaining my appearance”), and hubristic pride (e.g., “proud that I am an attractive person”) on a 5-point Likert-type scale ranging from 1 (*never*) to 5 (*always*). Subscales were computed by averaging responses across the four items for each emotion, with higher scores reflecting higher levels of the emotion. Evidence of validity and reliability has been reported previously with non-cancer AYAs (Castonguay et al., 2014; Chiminazzo et al., 2021).

##### Fitness-Related Self-Conscious Emotions

Participants completed the 16-item Body-Related Emotions in Fitness Instrument (BSE-FIT; Castonguay et al., 2016) to assess fitness-related shame, guilt, authentic pride, and hubristic pride. Participants were asked to indicate how often, on average, they experienced fitness-related shame (e.g., “ashamed that I am a person who is unfit”), guilt (e.g., “guilty that I do not do enough for my fitness”), authentic pride (e.g., “proud of my fitness efforts”), and hubristic pride (e.g., “proud of myself when I compare my fitness to others”) on a 5-point Likert-type scale ranging from 1 (*never*) to 5 (*always*). Subscales were computed by averaging responses across the four items for each emotion, with higher scores reflecting higher levels of the emotion. Evidence of validity and reliability has been reported previously with non-cancer AYAs (Castonguay et al., 2016; Pila et al., 2020).

#### Physical Activity

Self-reported PA was assessed using a modified version of the Godin Leisure-Time Exercise Questionnaire (GLTEQ; Godin and Shephard, 1985). Participants indicated how many times on average they engaged in mild (e.g., yoga, golf), moderate (e.g., fast walking, baseball), and vigorous (e.g., running, soccer) exercise for more than 15 min in a typical week. Aligned with the PA guidelines for cancer survivors [i.e., 90 min of moderate-to-vigorous PA (MVPA) per week; Campbell et al., 2019], we included the common modification of assessing the average duration (in hours and minutes) per session of each intensity category of PA. Aligning with previous research among non-cancer AYAs (Murray et al., 2021), respondents were asked to report the frequency and average duration of resistance exercise (e.g., free weights, bodyweight training). Resistance training was important since AYAs report a preference for resistance exercise (Adams et al., 2021) and qualitative evidence suggests AYAs may have unique experiences engaging in resistance training compared to aerobic PA (Vani et al., under review). Total scores for mild, moderate, and vigorous activity and resistance exercise were calculated by multiplying self-reported times per week by average duration of sessions for each type. In addition, moderate and vigorous scores were summed to yield a total MVPA score. This scale has been used in studies with AYAs (Brunet et al., 2018), has shown convergent validity with accelerometer measures in samples of cancer survivors (Amireault et al., 2015), and has demonstrated test-retest reliability (i.e., α-coefficient ranges from 0.46 to 0.96 in adolescent and adult samples; Godin and Shephard, 1985; Sallis et al., 1993).

### Data Analysis

Data were screened for outliers, missing data, and regression analysis assumptions prior to analyses (Tabachnick and Fidell, 2013). Preliminary analyses included descriptive statistics (e.g., mean, frequencies) to characterize the sample and the main study variables. Bivariate Pearson *r* and Spearman rho correlations were computed to test the relationships between variables using SPSS (Version 21).

To test the hypothesized associations among social support, body image, and PA, path analysis with maximum likelihood estimation was conducted using MPlus (Muthén and Muthén, 1998-2011). Two models were computed to examine appearance- and fitness-related emotions separately. The models were tested separately to examine the distinct influence of appearance and fitness contextualized emotions and to avoid measurement overlap and furthering model complexity. The models tested the direct path coefficients of: (i) social support (general and cancer-specific), *appearance*-related self-conscious emotions (shame, guilt, authentic pride, and hubristic pride), and PA (mild PA, MVPA, and resistance exercise); and (ii) social support, *fitness*-related self-conscious emotions, and PA. Indirect path coefficients of each model were computed for the effects of social support on PA through body-related emotions in both models. The following variables were set to correlate: (i) general and cancer-specific social support; (ii) shame, guilt, authentic pride, and hubristic pride; and (iii) mild PA, MVPA, and resistance exercise. Age, gender, and time since diagnosis were included as covariates in the models. Model goodness of fit was established using non-significant chi-square, and the accepted fit indices (Hu and Bentler, 1999) of: (i) Standardized Root Mean Square Residual (SRMR; values ≤0.08); (ii) Root Mean Square Error of Approximation (RMSEA; values close to 0.06); and (iii) Comparative Fit Index (CFI; values ≥0.90). Statistical significance was set to *p* < 0.05.

## Results

Data cleaning led to the removal of 85 records; some individuals opened the link but did not complete the consent form (*n* = 23), completed informed consent but did not begin the survey (*n* = 34), were identified as Bots^1^ (*n* = 20), began the survey multiple times (*n* = 4), or were not diagnosed with cancer between 15 and 39 years (*n* = 4). The remaining sample (*n* = 119) completed the electronic consent and survey, and was screened for missing data. Data were missing at the item level, and no item was missing greater than 5% of responses. Missing data were determined to be missing completely at random (MCAR χ^2^ = 703.42, *df* = 661, *p* > 0.05). Incomplete data were imputed using an Expectation Maximization algorithm (Dempster et al., 1977). Outliers were classified as values that deviated beyond ± 3 standard deviations from the mean and detected univariate outliers were winsorized (Osborne, 2010).

### Descriptive Results

Descriptive statistics for the analytical sample (*N* = 119) are presented in Table 1. AYAs were an average of 34.5 ± 5.5 years of age (range = 21–47), predominantly identified as White (82.4%), women (84.0%), married or living with a life partner (56.3%), diagnosed with stage II or III cancer (48.9%), and had completed cancer treatment (78.2%). Based on PA guidelines for cancer survivors (Campbell et al., 2019), 27.7% of AYAs reported meeting guidelines of both MVPA and resistance training. Descriptive statistics, bivariate correlations, and internal consistency for the main study variables and covariates are presented in Table 2. In general, participants reported social support and body-related emotion scores around the mid-point of the respective scales. Overall, higher scores for shame and guilt were reported compared to authentic and hubristic pride, and fitness emotion scores were generally higher than appearance emotions.

**TABLE 1:** Participant descriptive characteristics (*n* = 119).

Descriptive and study variables	*M* ± *SD* or *n* (%)
Age, years	34.5 ± 5.5
**Gender**	
Man	16 (13.4%)
Woman	100 (84.0%)
Other^a^	3 (2.5%)
Sexual orientation*	
Bisexual	7 (5.9%)
Gay	3 (2.5%)
Heterosexual	91 (76.5%)
Lesbian	1 (0.8%)
Pansexual	3 (2.5%)
Other^b^	8 (6.7%)
Prefer not to answer	5 (4.2%)
**Ethnicity^‡^**	
Arab	1 (0.8%)
Black	3 (2.5%)
Chinese	2 (1.7%)
Filipino	2 (1.7%)
Latin American/Hispanic	3 (2.5%)
South Asian	7 (5.9%)
White	98 (82.4%)
**Education^†^**	
High school	4 (3.4%)
College/Technical	12 (10.1%)
University undergraduate	53 (44.5%)
Post-graduate	47 (39.5%)
None of the above	1 (0.8%)
Relationship status*	
Single	33 (27.7%)
In a relationship, not living with partner	12 (10.1%)
Married/living with life partner	67 (56.3%)
Separated/Divorced	4 (3.4%)
Widowed	2 (1.7%)
Children (*n*, % yes at least one)	40 (33.6%)
Population size*	
Small (1,000–29,999)	15 (12.6%)
Medium (30,000–99,999)	25 (21.0%)
Large (100,000 +)	78 (66.1%)
Socioeconomic status (income)^§^	94,910 ± 63,839
**Cancer type^c^**	
Breast	39 (32.8%)
Hodgkin lymphoma	15 (12.6%)
Non-Hodgkin lymphoma	12 (10.1%)
Colorectal	7 (5.9%)
Ovarian	6 (5.0%)
Acute lymphoblastic leukemia	5 (4.2%)
**Stage of cancer at diagnosis^d^**	
0	3 (2.5%)
I	22 (18.5%)
II	33 (27.7%)
III	24 (20.2%)
IV	16 (13.4%)
Currently on treatment (*n*, % yes)*	26 (21.8%)
Number of treatments received	2.3 ± 1.0
**Type of treatment (*n*, % yes)^e^**	
Chemotherapy	94 (79.0%)
Surgery	79 (66.4%)
Radiation	60 (50.4%)
Hormone therapy	15 (12.6%)
Stem cell transplant	8 (6.7%)
Immunotherapy	7 (5.9%)
Time since diagnosis, years	4.2 ± 3.7
Time since treatment, years	3.0 ± 3.8
Met aerobic physical activity guidelines^f^	84 (70.6%)
Met resistance exercise guidelines^f^	41 (34.5%)
Met aerobic and resistance guidelines^f^	33 (27.7%)

*^a^Other consists of the following self-identified genders: transgender (n = 1), gender fluid (n = 1), and gender queer (n = 1). These individuals (n = 3) were removed from gender in the model.*

*^b^Other consists of the following self-identified sexual orientations: queer (n = 4), asexual (n = 2), questioning (n = 1), did not specify their sexual orientation (n = 1).*

*^c^Other cancer types included n = 4: thyroid, skin, and brain; n = 3: Ewing sarcoma; n = 2: acute myeloid leukemia, chronic myelogenous leukemia, and kidney; n = 1: appendix, bladder, cervical, laryngeal, lung, multiple myeloma, sarcoma, testicular, tongue, and uterine.*

*^d^Remaining 17.6% of AYAs did not know their cancer stage or reported that stage was not applicable.*

*^e^Multiple responses given.*

*^f^Meeting combined physical activity guidelines is defined as 90 min of at least moderate-intensity aerobic activity per week and two sessions of resistance exercise per week (Campbell et al., 2019). Using prior physical activity guidelines (i.e., 150 min of at least moderate-intensity aerobic activity per week; Schmitz et al., 2010), 48.7% met aerobic guidelines and 18.5% met aerobic and resistance guidelines.*

*n = 118. ^†^n = 117. ^‡^n = 116. ^§^n = 106.

**TABLE 2:** Descriptive statistics, bivariate correlations, and internal consistency for the main study variables (*n* = 119).

	1	2	3	4	5	6	7	8	9	10	11	12	13	14	15	16
1. Appearance shame^a^	–															
2. Appearance guilt^a^	0.76**	–														
3. Appearance authentic pride	−0.39**	−0.40**	–													
4. Appearance hubristic pride	−0.37**	−0.34**	0.61**	–												
5. Fitness shame^a^	0.74**	0.79**	−0.42**	−0.33**	–											
6. Fitness guilt^a^	0.58**	0.78**	−0.41**	−0.26**	0.82**	–										
7. Fitness authentic pride	−0.44**	−0.55**	0.57**	0.37**	−0.62**	−0.64**	–									
8. Fitness hubristic pride	−0.42**	−0.51**	0.55**	0.59**	−0.54**	−0.50**	0.79**	–								
9. General social support	−0.32**	−0.20*	0.19*	0.27**	−0.31**	−0.12	0.17	0.22*	–							
10. Cancer social support	−0.17	−0.10	0.01	0.07	−0.13	−0.16	0.21*	0.22*	0.37**	–						
11. Mild PA	−0.03	−0.15	0.07	0.04	−0.21*	−0.19*	0.08	0.11	0.17	0.22*	–					
12. MVPA	−0.12	−0.32**	0.24**	0.06	−0.36**	−0.37**	0.40**	0.30*	0.04	0.25**	0.25**	–				
13. Resistance exercise	−0.19*	−0.40**	0.39**	0.29**	−0.33**	−0.48**	0.48**	0.46**	0.02	0.05	0.10	0.28**	–			
14. Age	0.01	−0.00	−0.18	−0.17	−0.07	−0.09	0.02	0.00	−0.09	0.26**	0.13	0.06	−0.10	–		
15. Time since diagnosis^b^	−0.11	−0.18	−0.03	−0.04	−0.16	−0.19*	0.09	0.08	−0.19*	−0.02	0.04	0.14	0.04	0.20*	–	
16. Gender^c^	0.22*	0.30**	−0.07	−0.23*	0.19*	0.25**	−0.11	−0.18	0.04	−0.01	0.12	−0.11	−0.09	0.11	0.07	–
Mean	2.7	2.8	2.2	1.9	2.9	3.1	2.6	2.0	2.1	2.2	139.3	174.8	36.9	34.5	4.2	–
Standard deviation	1.1	0.9	0.8	0.8	1.0	1.1	1.1	1.0	0.7	1.2	125.5	152.4	57.7	5.5	3.7	–
Score range^d^	1–5	1–5	1–5	1–4	1–5	1–5	1–5	1–5	0–4	0–4	0–483	0–587	0–240	21–47	0–19	–
Internal consistency (α)	0.92	0.84	0.86	0.88	0.87	0.94	0.94	0.90	0.87	0.74	–	–	–	–	–	–

*PA = physical activity; MVPA = moderate-to-vigorous physical activity; α = Cronbach’s alpha.*

*^a^Significant (p < 0.05) bivariate correlations for guilt-free shame (GFS), shame-free guilt (SFG), and the remaining model variables included: (i) appearance GFS with general social support (r = −0.26) and MVPA (r = 0.19), (ii) fitness GFS with appearance hubristic pride (r = −0.21), fitness hubristic pride (r = −0.23), and general social support (r = −0.37), (iii) appearance SFG with fitness authentic pride (r = −0.33) and hubristic pride (r = −0.30), mild PA (r = −0.19), MVPA (r = −0.35), resistance exercise (r = −0.39), and gender (r_s_ = 0.21), and (iv) fitness SFG with fitness authentic pride (r = −0.24), general social support (r = 0.24), and resistance exercise (r = −0.36).*

*^b^Time since diagnosis measured in years.*

*^c^Spearman rho correlation coefficients; coded as 1 = man; 2 = woman; n = 116.*

*^d^Scale range for body-related emotions is 1–5 and social support is 0–4.*

*p < 0.05, **p < 0.001.

### Main Results

Preliminary models produced spurious relationships involving appearance- and fitness-related shame and guilt, and the correlations between shame and guilt were significant (*r* = 0.76–0.82, *p* < 0.001). To address a potential model misfit, revised models using guilt-free shame (GFS) and shame-free guilt (SFG) were tested. Based on previous research (Sabiston et al., 2010), GFS was assessed as the standardized residual associated with predicting shame from guilt (operationalized as maladaptive negative emotion focused on the self; Tangney and Dearing, 2002), while SFG was assessed as the standardized residual associated with predicting guilt from shame (operationalized as a behavior-focused and often adaptive negative emotion without conflating the negative value of the self; Tangney and Dearing, 2002). Significant (*p* < 0.05) bivariate correlations for GFS, SFG, and the remaining model variables are presented under Table 2.

#### Appearance-Related Emotions Model

**FIGURE 1:**
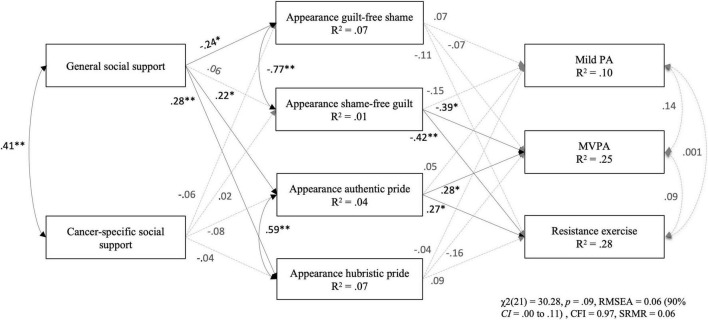
Standardized path coefficients and explained variance of the model in predicting social support, appearance-related emotions, and physical activity. Note. Covariates included in the model are age, gender, and time since diagnosis. Variance explained for each endogenous variable are reported within the observed variable boxes. None of the indirect effects were significant. PA = physical activity; MVPA = moderate-to-vigorous physical activity. *p < 0.05; **p < 0.001.

The results of the path analysis are presented in Figure 1. Goodness-of-fit statistics were: χ^2^(21) = 30.28, *p* = 0.09, RMSEA = 0.06 (90% CI = 0.00–0.11), CFI = 0.97, and SRMR = 0.06. Significant (*p* < 0.05) direct path coefficients included: general social support to appearance shame, authentic pride, and hubristic pride, appearance guilt to MVPA and resistance exercise, and appearance authentic pride to MVPA and resistance exercise (standardized coefficients presented in Figure 1). In addition, cancer-specific social support was positively associated with mild PA (β = 0.19, *p* < 0.05) and MVPA (β = 0.29, *p* < 0.001). Overall, the model predicted 10% of the variance in mild PA, 25% in MVPA, and 28% in resistance exercise.

#### Fitness-Related Emotions Model

**FIGURE 2:**
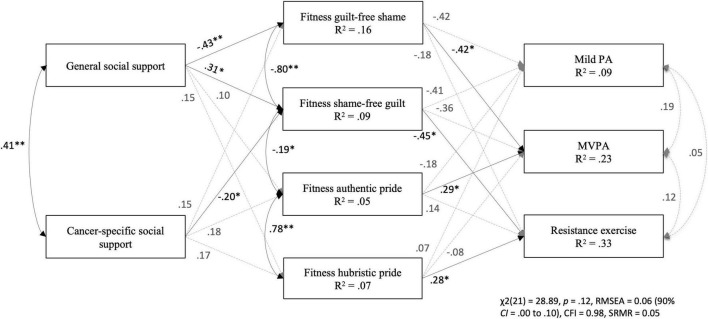
Standardized path coefficients and explained variance of the model in predicting social support, fitness-related emotions, and physical activity. Note. Covariates included in the model are age, gender, and time since diagnosis. Variance explained for each endogenous variable are reported within the observed variable boxes. None of the indirect effects were significant. PA = physical activity; MVPA = moderate-to-vigorous physical activity. *p < 0.05; **p < 0.001.

The results of the path analysis are presented in Figure 2. Goodness-of-fit statistics were: χ^2^(21) = 28.89, *p* = 0.12, RMSEA = 0.06 (90% CI = 0.00–0.10), CFI = 0.98, and SRMR = 0.05. Significant (*p* < 0.05) direct path coefficients included: general social support to fitness shame and guilt, cancer-specific social support to fitness guilt, fitness shame and authentic pride to MVPA, and fitness guilt and hubristic pride to resistance exercise (standardized coefficients presented in Figure 2). In addition, cancer-specific social support was positively associated with mild PA (β = 0.18, *p* < 0.05) and MVPA (β = 0.22, *p* < 0.05). Overall, the model predicted 9% of the variance in mild PA, 23% in MVPA, and 33% in resistance exercise.

## Discussion

The aim of this study was to test the relationships between social support, appearance- and fitness-related self-conscious emotions, and PA. Findings demonstrate that social support is associated with mild PA and MVPA, body-related emotions are associated with MVPA and resistance exercise, and social support is directly related to body-related emotions. However, there were no indirect effects of body-related emotions and the relationship between social support and PA. To further provide context to the findings, just over one quarter of the sample were meeting PA guidelines (Campbell et al., 2019) of 90 or more minutes of MVPA and at least two resistance-training sessions per week. The results have implications for further testing these relationships over time and provide preliminary support for developing strategies aimed at increasing social support and improving body image for AYAs during and after treatment for cancer.

In partial support of the hypothesis, greater perception of cancer-specific social support was associated with increased levels of mild PA and MVPA. Although AYAs reported similar levels of general and cancer-specific social support, general social support was not associated with PA. Previous literature with AYAs found that support group involvement, but not general social support, moderated the association between stress and PA (Brunet et al., 2014). Given that group PA with other cancer survivors has shown to be useful in increasing PA confidence (Zebrack et al., 2017; Pugh et al., 2021) and behavior (Gill et al., 2016), it may be important to further examine the aspects of cancer-specific support that may be most beneficial for increasing PA levels among AYAs. It may be valuable to explore the (i) types of cancer-specific support (e.g., emotional, informational), (ii) characteristics of individual(s) involved (e.g., similar-aged/-life stage peer(s), mentor(s) further out from treatment), (iii) methods of connecting (e.g., virtual, in-person), and (iv) contexts (e.g., conversations, exercising together). Further, age was positively associated with cancer-specific social support, indicating that those who are older have higher perceptions that others understand what they are going through. And so, particular attention toward adolescents and the provision of cancer-specific support may be needed.

Aligned with sociocultural perspectives (Petrie and Greenleaf, 2012) and previous literature (Larouche and Chin-Peuckert, 2006; Williamson et al., 2010) and consistent with hypotheses, social support was associated with numerous body-related emotions. The current findings suggest that those who perceive high general support from others report experiencing higher appearance-related authentic and hubristic pride, and lower appearance and fitness GFS, yet higher fitness SFG. Given that literature demonstrates that shame thwarts PA, while guilt can encourage individuals to initiate PA (Sabiston et al., 2010; Castonguay et al., 2015, 2017), these latter findings are not entirely troubling. However, it is important to understand the aspects of general social support (e.g., types, individuals providing support) that increase feelings of fitness-related guilt. Further, greater perception of cancer-specific social support was associated with lower fitness-related SFG, indicating that receiving support from those who validate one’s cancer experience may make AYAs feel less alone in their challenges with fitness, and therefore, reduces one’s guilt toward their fitness. Taken together, these results suggest that a positive perception of a supportive social network can help AYAs to feel more positive and less negative body-related self-conscious emotions. Thus, it may be important to ensure AYAs feel socially supported during and after treatment. Given that time since diagnosis was negatively associated with general social support, those who are further out from diagnosis may require additional ongoing supportive care.

In support of hypotheses, appearance- and fitness- related emotions were associated with PA. These findings are generally consistent with other cross-sectional studies among women (Sabiston et al., 2010; Castonguay et al., 2017; Pila et al., 2020) and men (Castonguay et al., 2015; Gilchrist et al., 2018). Overall, the fitness-related emotions were more consistently related to MVPA and resistance exercise, while only appearance-related guilt and authentic pride were related to PA. Intervening on fitness-related emotions may be ideal for enhancing PA among AYAs. More attention on fitness-related emotions in research, theory, and practice is needed. Notably, however, the body-related emotions were not related to mild PA, which may indicate that body-related feelings may not play a role in engagement in light and lifestyle activities (e.g., walking). Previous work in this area does not tease out PA into different intensities, yet some qualitative evidence suggests that different types of PA may be important outcomes of body image (Vani et al., under review) and should be further explored in future research.

Higher reports of body appearance and fitness-related guilt and shame were related to lower levels of MVPA and resistance exercise. These findings are consistent with theoretical tenets (e.g., self-objectification theory; Fredrickson and Roberts, 1997) and general body image literature among AYAs (Wu et al., 2015; Vani et al., 2021) wherein negative body image can be a barrier for PA engagement. The results extend previous findings by exploring appearance and functional facets of affective body image among AYAs. Although prior research with adult breast cancer survivors found appearance-related shame was related to MVPA (Castonguay et al., 2017), the current study’s findings demonstrated that fitness-related, but not appearance-related, GFS was related to MVPA. Therefore, AYAs who blame themselves for their fitness may have an especially difficult time engaging in activity after a cancer diagnosis. Given that shame is a particularly painful self-conscious emotion (Tracy and Robins, 2007), strategies to reduce fitness-related shame are needed. Cognitive dissonance-based psychoeducation related to challenging societal expectations for fitness and body ideals may help to minimize fitness-related shame (Smith and Petrie, 2008).

In contrast to previous work that demonstrates guilt as a reparative emotion with positive effects on PA (Sabiston et al., 2010; Castonguay et al., 2017), appearance-related SFG was negatively associated with MVPA and resistance exercise, and fitness-related SFG was negatively associated with resistance exercise. These results are aligned with descriptions of the maladaptive aspects of this emotion (Tangney and Tracy, 2012). AYAs who express guilt over their appearance and fitness efforts are less likely to engage in intentional aerobic and resistance activities, indicating a need for the use of guilt-reducing strategies. Self-compassion approaches, such as journal prompts or expressing gratitude to the self and body-related efforts, may be useful to express emotions and alleviate feelings of body shame and guilt (Mosewich et al., 2011). Future research is needed to examine these relationships over time.

Experiencing positive body-related emotions was related to engagement in greater levels of aerobic activity and resistance exercise. Overall, these findings support the important role of positive body image in facilitating health-promoting behavior (Tylka and Wood-Barcalow, 2015; Gilchrist et al., 2018). In particular and aligned with previous literature (Sabiston et al., 2010; Castonguay et al., 2015), appearance- and fitness-related authentic pride were associated with MVPA, while appearance-related authentic pride was also related to resistance exercise. AYAs may use PA as a way of achieving their appearance- and fitness-related goals (Castonguay et al., 2013). Given these findings, it would be advantageous to enhance AYAs’ appearance- and fitness-related authentic pride. Strategies to achieve this may include facilitated interventions wherein AYAs are prompted to connect their effort, competence, and manageable goal setting to their PA achievements (Castonguay et al., 2015).

Furthermore, aligned with previous research (Gilchrist et al., 2018), fitness-related, but not appearance-related, hubristic pride (related to feeling superior to others’ fitness), translated to higher engagement in resistance exercise. AYAs may use resistance exercise to display their superior fitness to others (Castonguay et al., 2013). Although fitness-related hubristic pride is associated with health-enhancing behavior, it should be noted that hubristic pride has been related to worsened mental health (Tracy et al., 2009), and therefore should not be promoted to foster PA maintenance with AYAs. It would be worthwhile to explore these relationships over time and uncover the mechanisms through which the facets of pride facilitate increased aerobic and resistance activity.

Contrary to our hypotheses, there were no indirect effects of body-related emotions and the relationship between social support and PA. The tested direction of effects is supported by conceptual and theoretical notions and prior research (Sabiston et al., 2010; Petrie and Greenleaf, 2012; Castonguay et al., 2015), however, there are no specific models within the oncology context to link social support, body image, and PA. Based on the current cross-sectional findings, future research efforts could focus on a more nuanced understanding of cancer-related support, assess other dimensions of body image (perceptual, cognitive, and behavioral) and body image affect indicators (envy, embarrassment), and test the relationships over time.

### Study Limitations and Future Directions

Findings should be interpreted in context with the following limitations. The cross-sectional nature of this study precludes our understanding of directionality of the associations, and causal inferences cannot be made. Although there is conceptual and empirical evidence for the direction of relationships tested and discussed (Sabiston et al., 2010; Petrie and Greenleaf, 2012; Castonguay et al., 2015, 2017), reverse or bidirectional associations may also be reasonable and should therefore be tested in future research. Further, measurement and structural models were not tested due to sample size, and so future research should examine these relationships using structural equation modeling. In addition, due to the homogeneity of the sample (e.g., mainly educated, White women), generalizability is limited. Future research efforts should be aimed at recruiting a larger and more diverse sample of AYAs. Moreover, body image may differ based on demographic considerations. Whilst the current study controlled for age and time since diagnosis in the path models, the range of age and time since diagnosis were broad. Thus, future research should consider assessing body image differences based on age groups, developmental stages, and cancer trajectory. Further, the survey was collected during the COVID-19 pandemic, and with widespread restrictions, engagement in PA and social support and body image scores may have been affected. Future research should explore these relationships among AYAs post-pandemic. Finally, PA was assessed using a self-report measure, which can have measurement limitations (Schmidt et al., 2008). Assessing the associations using an objective PA measure (e.g., accelerometers) warrants future investigation.

## Conclusion

The current study uniquely explored appearance- and fitness-related body image emotions and examined both aerobic activity and resistance exercise among AYAs. The findings underscore the importance of assessing appearance- and fitness-related self-conscious emotions. This study is strengthened by the inclusion of cancer-specific social support, mild PA, and resistance exercise, which are not well understood among AYAs. The results underscore the importance of the development and use of supportive care initiatives and strategies aimed at improving social support (e.g., facilitate the provision of general and cancer-specific social support) and body image (e.g., psychoeducation, compassion-focused, and goal-achievement interventions) for potential increases in PA participation.

## Data Availability Statement

The original contributions presented in the study are included in the article/supplementary material, further inquiries can be directed to the corresponding author.

## Ethics Statement

The studies involving human participants were reviewed and approved by the University of Toronto Research Ethics Board. The patients/participants provided their written informed consent to participate in this study.

## Author Contributions

CS, DS, LT, and MV: conception and design and reviewing the manuscript. MV and CS: acquisition and interpretation of the data. MV: drafting the manuscript. All authors contributed to the article and approved the submitted version.

## Conflict of Interest

The authors declare that the research was conducted in the absence of any commercial or financial relationships that could be construed as a potential conflict of interest.

## Publisher’s Note

All claims expressed in this article are solely those of the authors and do not necessarily represent those of their affiliated organizations, or those of the publisher, the editors and the reviewers. Any product that may be evaluated in this article, or claim that may be made by its manufacturer, is not guaranteed or endorsed by the publisher.

## Footnotes

1 Bots were identified in consultation with the Information Technology department at the authors’ university. Identification occurred using a combination of email address, time to complete the survey, and responses provided. Using the same approach, the remaining participants were deemed genuine.
